# Symptom Burden among Older COVID-19 Survivors Two Years after Hospital Discharge

**DOI:** 10.14336/AD.2023.0304

**Published:** 2023-12-01

**Authors:** Ying Zhang, Zhaojie Han, Yang Dai, Yuhui Liu, Qinghua Wang, Lixia Cheng, Chuyue Xiong, Chao Hou, Xinyue Yang, Yidan Ye, Qian Zhao, Naifu Nie, Xiangyu Ma, Huan Tang, Anqiang Zhang, Zhenhong Hu, Guoqiang Cao, Paul Jones, Jianxin Jiang, Ling Zeng, Yong He, Li Li

**Affiliations:** ^1^Department of Respiratory Medicine, Daping Hospital, Third Military Medical University (Army Medical University), Chongqing, China.; ^2^Department of Thoracic Surgery, Southwest Hospital, Third Military Medical University (Army Medical University), Chongqing, China.; ^3^Department of Neurology and Centre for Clinical Neuroscience, Daping Hospital, Third Military Medical University (Army Medical University), Chongqing, China.; ^4^Wuhan Huoshenshan Hospital, Wuhan, China.; ^5^Department of Medical and Research Management, Daping Hospital, Third Military Medical University (Army Medical University), Chongqing, China.; ^6^Taikang Tongji Hospital, Wuhan, China.; ^7^Department of Epidemiology, College of Preventive Medicine, Third Military Medical University (Army Medical University), Chongqing, China.; ^8^Department of Trauma Medical Center, Daping Hospital, State Key laboratory of Trauma, Burns, and Combined Injury, Third Military Medical University (Army Medical University), Chongqing, China.; ^9^Departmemt of Respiratory Medicine, Chinese PLA General Hospital of Central Theater Command, Wuhan, China.; ^10^Institute of Infection and Immunology, University of London, London, UK.; ^11^GlaxoSmithKline, Brentford, UK

**Keywords:** COVID-19, older, long COVID, symptom burden, SARS-CoV-2

## Abstract

To study the long-term symptom burden among older COVID-19 survivors 2 years after hospital discharge and identify associated risk factors. The current cohort study included COVID-19 survivors aged 60 years and above, who were discharged between February 12 and April 10, 2020, from two designated hospitals in Wuhan, China. All patients were contacted via telephone and completed a standardized questionnaire assessing self-reported symptoms, the Checklist Individual Strength (CIS)-fatigue subscale, and two subscales of the Hospital Anxiety and Depression Scale (HADS). Of the 1,212 patients surveyed, the median (IQR) age was 68.0 (64.0-72.0), and 586 (48.3%) were male. At the two-year follow-up, 259 patients (21.4%) still reported at least one symptom. The most frequently self-reported symptoms were fatigue, anxiety, and dyspnea. Fatigue or myalgia, which was the most common symptom cluster (11.8%; 143/1212), often co-occurred with anxiety and chest symptoms. A total of 89 patients (7.7%) had CIS-fatigue scores ≥ 27, with older age (odds ratio [OR], 1.08; 95% CI: 1.05-1.11, P < 0.001) and oxygen therapy (OR, 2.19; 95% CI: 1.06-4.50, P= 0.03) being risk factors. A total of 43 patients (3.8%) had HADS-Anxiety scores ≥ 8, and 130 patients (11.5%) had HADS-Depression scores ≥ 8. For the 59 patients (5.2%) who had HADS total scores ≥ 16, older age, serious illness during hospitalization and coexisting cerebrovascular diseases were risk factors. Cooccurring fatigue, anxiety, and chest symptoms, as well as depression, were mainly responsible for long-term symptom burden among older COVID-19 survivors 2 years after discharge.

As the global fight against COVID-19 has entered the third year, the global disease burden is still sharply increasing. As of August 23, 2022, more than 594 million confirmed cases and 6.4 million deaths have been reported (WHO dashboard - accessed 23/08/2022). Various multiorgan injuries and symptoms have been reported successively after patients surpass the acute stage of SARS-CoV-2 infection [1-3] and the duration of this disease remains to be uncertain. Patients with postinfection symptoms that occur 3 months after onset and last for at least 2 months without any other reasons are considered to have ‘long COVID’, as suggested by a Delphi consensus from the WHO Clinical Case Definition Working Group [4]. To date, a series of studies have conducted long-term follow-up examinations to determine health-related outcomes of COVID-19. Recently, in a large cohort study including a baseline characteristics-matched control group, COVID-19 survivors who had been discharged from the hospital for 2 years had a remarkably lower health status than the uninfected population [5], suggesting that ongoing follow-up is needed to better characterize the long-term effects of COVID-19.

Among patients with SARS-CoV-2 infection, older people are more severely affected. A systematic review of studies on the relationship between age and COVID-19-associated morbidity or mortality indicated that older adults have a higher susceptibility to symptomatic COVID-19 [6]. Besides, age was found to be correlated with increased risks of symptom persistence and long COVID burden, even 2 years after hospital discharge [5, 7, 8]. Nevertheless, no study has specifically examined the long-term health outcomes of older COVID-19 patients with a follow-up longer than 1 year after discharge [9]. Herein, the symptom burden among older COVID-19 survivors 2 years after hospital discharge was studied.

## MATERIALS AND METHODS

### Study design and patients

This prospective cohort study included older COVID-19 survivors who were discharged from Huoshenshan Hospital and Taikang Tongji Hospital in Wuhan, China from February 12 to April 10, 2020. All adults aged 60 years and above with laboratory-confirmed COVID-19 were screened for eligibility. The exclusion criteria included patients aged below 60 years, patients who died before the follow-up, and patients who declined to participate in the test or were unable to be contacted. The study was conducted in accordance with the *Strengthening the Reporting of Observational Studies in Epidemiology* (STROBE) reporting guidelines for cohort studies and was approved by the Ethics Committee of the Daping Hospital, Army Medical University (No. 202153). Verbal informed consent was obtained from all patients or their legal guardians prior to the survey for the telephone follow-up.

### Procedures

A telephone interview was carried out by trained medical staff. Participants were contacted in order of discharge dates in their medical records. All patients completed a standardized questionnaire of self-reported symptoms, which has been described in previous studies [8, 10]. Each symptom was rated with a 4-point Likert scale (no problems, mild problems, moderate problems, or severe problems) and symptoms were considered to be present if at least 1 problem was rated as moderate or severe. Then we used a two-step approach to realize symptom clustering. First, we identified strongly correlated self-reported symptoms by using exploratory polychoric factor analysis (using the oblimin rotation). To identify the ideal number of factors, we used “parallel” analysis. Second, we included each symptom into the cluster in which its factor loading was highest and made some adjustments to some clusters according to their clinical characteristics. In addition, due to common complaints of fatigue and the prevalences of anxiety and depression among COVID-19 survivors during follow-up [11, 12], patients also completed the Checklist Individual Strength (CIS)-fatigue subscale, and two Hospital Anxiety and Depression (HADS) subscales, i.e., the HADS-Anxiety (HADS-A) and the HADS-Depression (HADS-D). The CIS-fatigue subscale consists of 8 statements, with each statement rated on a scale from 1-7. Hence, the total CIS-fatigue scores range from 8 to 56, and a cutoff value of ≥27 is used to indicate abnormal fatigue [13]. For the HADS-A and HADS-D, each subscale consists of 7 questions with a 4-point Likert scale (0-3). The scores for each subscale range from 0 to 21 and a cutoff value of ≥ 8 indicates the presence of symptoms of anxiety or depression [14]. To measure the overall mental burden, scores from the HADS-A and HADS-D were simply summed as a whole to calculate the HADS total scores, and scores ≥ 16 indicated a heavier mental burden [15]. Participants who effectively took part in the assessment of the above scales were asked to answer each item according to how they felt or behaved in the past couple of months. Three attempts were made to contact those who did not respond to the telephone interview at first.

### Data acquisition

The disease severity was defined by the *World Health Organization guidelines for COVID-19* and was described in a previous study [16]. Baseline characteristics during hospitalization, including demographic characteristics (age, gender, and cigarette smoking), clinical features (coexisting disorders) and treatments (ICU admission, oxygen therapy and mechanical ventilation), were retrieved from electronic medical records. All data were double entered and verified using EpiData software (version 3.1, EpiData Association).

### Statistical analysis

The Kolmogorov-Smirnov test-was used to test the normality of the data. Continuous variables were expressed as median and interquartile range (IQR) and compared by the Mann-Whitney U test. Categorical variables were expressed as count (percentage) and were compared by Pearson’s χ^2^ test or Fisher’s exact test. The identified symptom clusters were visualized by a cooccurrence network using Gephi 0.9.2. To explore the potential risk factors for the occurrence of CIS scores ≥ 27 or HADS scores ≥ 16, a univariate logistic regression model was utilized to identify potential risk factors with *P* < 0.10, which was then adjusted by a stepwise (forward likelihood ratio) selection process in a multivariable logistic regression model, whereas age, gender, and disease severity were forced into the model considering their clinical importance. All tests were 2-sided, with P < 0.05 considered significant. Data were analyzed with SPSS version 26.0 for Windows (IBM SPSS Statistics) and R statistical software version 4.1.1 (R Project for Statistical Computing).

**Table 1:** Characteristics of enrolled participants.

Characteristics	Patients, No. (%)			*P* value		

	Enrolled (N=1212)	Severe disease (N=422)	Nonsevere disease (N=790)	Lost to follow-up (N=943)	Enrolled vs. lost to follow-up	Severe vs. nonsevere
Age - Median (IQR), years^a^	68.0(64.0-72.0)	70.0(65.0-74.3)	67.0(64.0-71.3)	70.0(65.0-78.0)	<0.001	<0.001
Sex - No. (%)						
Male	586(48.3)	234(55.5)	352(44.6)	468(49.6)	0.56	<0.001
Female	626(51.7)	188(44.5)	438(55.4)	475(50.4)		
Cigarette smoking - No. (%)					0.19	0.61
Never	1104(91.1)	388(92.0)	716(90.6)	871(92.4)		
Former	16(1.3)	4(0.9)	12(1.5)	17(1.8)		
Active	92(7.6)	30(7.1)	62(7.9)	55(5.8)		
Coexisting disorders number					0.11	<0.001
0	506(41.8)	147(34.8)	359(45.4)	360(38.2)		
1	369(30.4)	126(29.9)	243(30.8)	284(30.1)		
≥2	337(27.8)	149(35.3)	188(23.8)	299(31.7)		
Hypertension	514(42.4)	211(50.0)	303(38.4)	419(44.4)	0.35	<0.001
Diabetes mellitus	234(19.3)	97(23.0)	137(17.3)	173(18.3)	0.57	0.02
Cardiovascular diseases	189(15.6)	84(19.9)	105(13.3)	166(17.6)	0.21	0.002
Chronic liver diseases	51(4.2)	17(4.0)	34(4.3)	46(4.9)	0.46	0.82
Cerebrovascular diseases	64(5.3)	28(6.6)	36(4.6)	94(10.0)	<0.001	0.12
Chronic kidney diseases	28(2.3)	11(2.6)	17(2.2)	40(4.2)	0.01	0.62
Tumor	35(2.9)	17(4.0)	18(2.3)	31(3.3)	0.59	0.08
Tracheitis	33(2.7)	15(3.6)	18(2.3)	35(3.7)	0.19	0.19
COPD	20(1.7)	8(1.9)	12(1.5)	26(2.8)	0.08	0.62
Length of hospital stay, days^a^	15.0(10.0-22.0)	16.0(10.0-24.0)	15.0(10.0-21.0)	14.0(9.0-21.0)	0.06	0.001
Follow-up time, days^a^	736(720-762)	729(714-755)	739(724-768)	-	-	<0.001
ICU - No. (%)	40(3.3)	36(8.5)	4(0.5)	47(5.0)	0.05	<0.001
Oxygen therapy, No. (%)	953(78.6)	389(92.2)	564(71.4)	752(79.7)	0.53	<0.001
Mechanical ventilation, No. (%)	21(1.7)	20(4.7)	1(0.1)	24(2.5)	0.19	<0.001

Abbreviation: IQR, Inter-quartile range; ICU, Intensive Care Unit; COPD, Chronic Obstructive Pulmonary Disease; NA, not applicable.

a Statistical tests were calculated with the Mann-Whitney U test. The rest: Pearson’s χ^2^ test.

## RESULTS

### Patient characteristics

**Figure 1.**
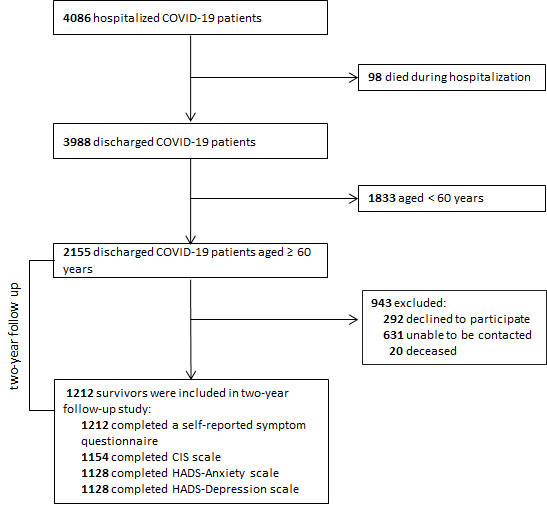
Study flowchart.

Of 2,155 discharged older adults with COVID-19, 1,212 survivors (56.3%) who were available for the 2-year interview were included in the final analysis. The median (IQR) age of the participants was 68.0 (64.0-72.0) years old (range, 60-94 years old), and 586 (48.3%) were male. In total, 1,212 participants completed a symptom questionnaire, 1,154 participants completed the CIS scale, and 1,128 participants completed the two HADS subscales (Fig. 1). The median (IQR) time from discharge to the 2-year follow-up was 736 (720-762) days. The median (IQR) duration of hospital stay was 15.0 (10.0-22.0) days. During hospitalization, 953 patients (78.6%) received oxygen therapy, among whom 21 patients (1.7%) received mechanical ventilation, and 40 patients (3.3%) were admitted to the intensive care unit (ICU). Altogether, 422 patients (34.8%) were categorized as having severe disease. Patients in the severe group were older, with proportionally more male individuals, a higher percentage of coexisting disorders, ICU admission, oxygen therapy and mechanical ventilation, and longer length of hospital stay (Table 1). Compared with enrolled patients, those who were lost to receive follow-up (N=943) were older and presented a higher percentage of coexisting cerebrovascular diseases and chronic kidney diseases. No significant difference was found in terms of gender or cigarette smoking (Table 1).

### Long-term symptoms at the two-year follow-up

At 2 years after discharge, 259 survivors aged 60 years and above (21.4%) still reported at least one symptom, and the percentage was higher in patients with severe disease during hospitalization (severe vs. nonsevere: 25.4% vs. 19.2%; OR, 1.43; 95% CI: 1.08-1.89; *P* = 0.01) (Supplementary Table 1). Of those with persisting symptoms, 161 (13.3%) reported 1 symptom, 58 (4.8%) reported 2 symptoms, and 40 (3.3%) reported 3 or more symptoms. The most common symptom was fatigue (9.3%), followed by anxiety (7.4%), dyspnea (2.5%), myalgia (2.5%), and chest tightness (2.2%). The prevalence of anxiety in the severe group was significantly higher than that in the nonsevere group (severe vs. nonsevere: 10.2% vs. 5.9%; OR, 1.79; 95% CI: 1.17-2.76; *P* = 0.01).

**Figure 2.**
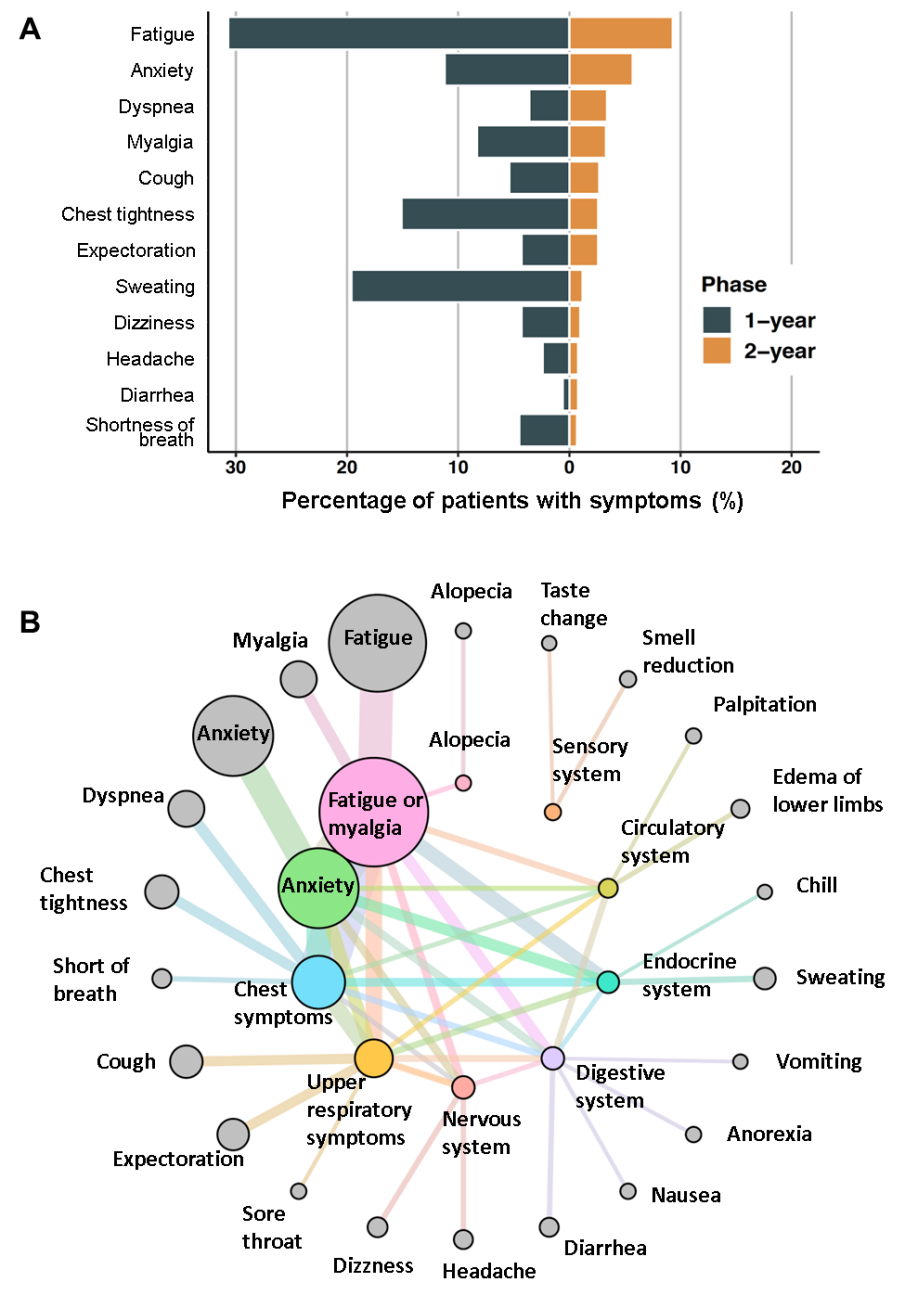
Long-term symptom patterns among older COVID-19 survivors. (A) Percentage of older patients presenting with COVID-19-related symptoms at the 1-year and 2-year follow-up; (B) Cooccurrence network of symptom clusters 2 years after hospital discharge. Outer circles represent individual symptoms. The outer circle area represents the proportion of participants with that symptom. These are linked to inner circles, which represent symptom clusters. The width of the link lines also represents the proportion of patients with that symptom. The circle area for clusters represents the proportion of participants with at least one symptom from that cluster. Central link lines between symptom clusters represent the cooccurrence of symptom clusters. Line width represents the degree of co-occurrence.

A total of 890 COVID-19 survivors aged 60 years and above participated in both one-year and two-year follow-up visits on symptom dynamics in a recent study [8]. Among these survivors, a prominent decreasing trend was found in most symptoms. However, the percentage of participants who reported dyspnea did not change significantly (one-year vs. two-year; 3.6% [32 patients] vs. 3.4% [30 patients]). In addition, the occurrence of anxiety exhibited a significant decrease in the overall population (one-year vs. two-year: 11.2% [100 patients] vs. 5.7% [51 patients]; difference, 5.5%; 95% CI: 2.9%-8.1%; *P* < 0.001) and in the nonsevere subgroup (one-year vs. two-year: 10.4% [59 patients] vs 4.0% [23 patients]; difference,6.4%; 95%CI: 3.4%-9.3%; *P* < 0.001). There was a nonsignificant decrease observed in the severe subgroup (one-year vs. two-year: 12.7% [41 patients] vs. 8.7% [28 patients]) (Fig. 2A, full details in Supplementary Table 2).

In view of the relatively low prevalence rate and dispersed self-reported symptoms, we further explored symptom clustering for a more concentrated pattern of health effects. We found that several of the 22 self-reported symptoms were strongly correlated and could be combined into 10 symptom clusters (Fig. 2B). The most common symptom cluster among participants was “fatigue or myalgia”, with a prevalence of 11.8% (143/1212), followed by “anxiety” (7.4%; 90/1212) and “chest symptoms” (5.3%; 64/1212). We also set our sights on co-occurrence between clusters. Notably, long-term smell and taste disorders were shown to be independent of other symptom clusters. Fatigue or myalgia, as the most prevalent symptom cluster, frequently co-occurred with anxiety and chest symptoms and was the only cluster to co-occur with alopecia. In addition, the first three clusters often co-occurred in a relatively high proportion.

### CIS-fatigue scores at the two-year follow-up

**Figure 3.**
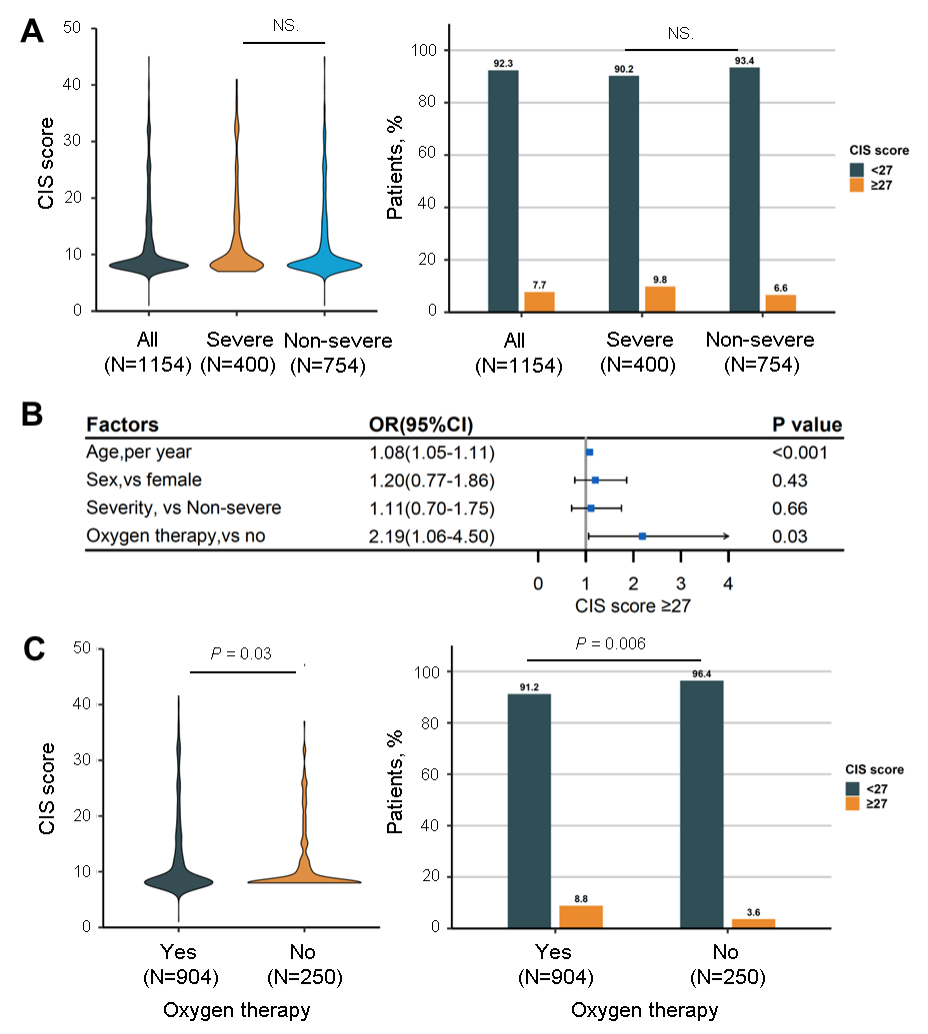
CIS-fatigue scores among older COVID-19 survivors. (A) CIS scores (Mann-Whitney U test) at 2 years after hospital discharge. Lines within bars denote medians, and error bars denote 95% CIs (left). Percentage of patients with CIS scores ≥ 27 or < 27 (Pearson’s χ^2^ test, right); (B) Factors associated with CIS scores ≥27; (C) CIS scores (Mann-Whitney U test) and percentage of patients with scores ≥27 or <27 (Pearson’s χ^2^ test) in those receiving oxygen therapy or not. CIS, Checklist Individual Strength; OR, odds ratio. The severe group was defined as those with fever or suspected respiratory infection, plus one of the following conditions: respiratory rate more than 30 breaths per minute; severe respiratory distress; or oxygen saturation as measured by pulse oximetry less than or equal to 93% on room air.

At the two-year follow-up, the median (IQR) score of CIS-fatigue was 8 (8-14) and 89 patients (7.7%) had scores of 27 or higher. Patients with severe disease had a nonsignificantly higher median (IQR) CIS-fatigue score (severe vs. nonsevere: 8 [8-15] vs. 8 [8-13]) and a higher proportion of patients with the CIS-fatigue score of at least 27 (severe vs. nonsevere: 9.8% [39 patients] vs. 6.6% [50 patients]) (Fig. 3A).

Univariate analysis revealed that age, oxygen therapy, former cigarette smoking, and coexisting cerebrovascular diseases or chronic obstructive pulmonary diseases were linked with increased risks of CIS scores of 27 or higher. After multivariable adjustment, only age (OR, 1.08; 95% CI: 1.05-1.11, *P* < 0.001) and oxygen therapy (OR, 2.19; 95% CI: 1.06-4.50, *P*=0.03) were risk factors (Fig. 3B and Supplementary Table 3). Patients who received oxygen therapy had a markedly higher median (IQR) CIS-fatigue score (Yes vs. No: 8 [8-14] vs. 8 [8-11.3], *P* = 0.03) as well as a higher proportion of patients with a CIS-fatigue score of at least 27 (Yes vs. No: 8.8% [80 patients] vs. 3.6% [9 patients], *P* = 0.006) (Fig. 3C).

**Figure 4.**
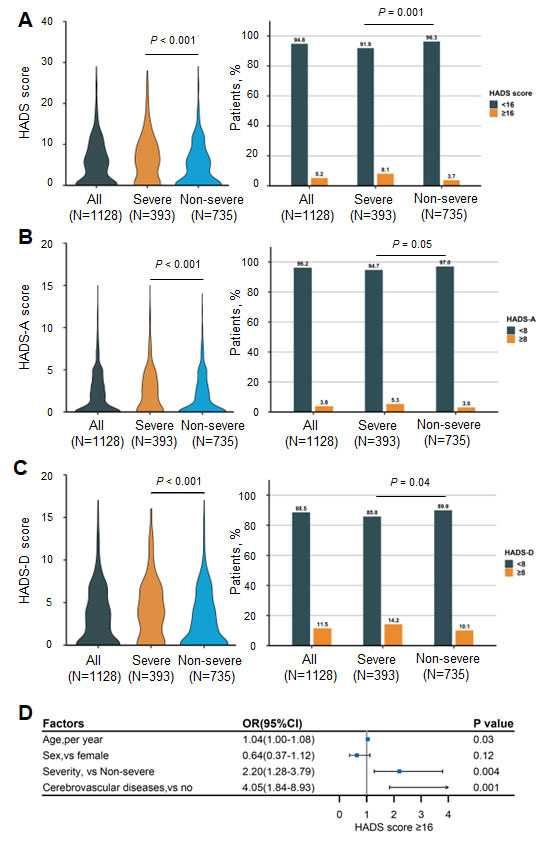
HADS scales among older COVID-19 survivors. (A) HADS total scores (Mann-Whitney U test) and percentage of patients with scores ≥ 16 or < 16 (Pearson’s χ^2^ test). Lines within bars denote medians, and error bars denote 95% CIs. (B) HADS-A scores (Mann-Whitney U test) and percentage of patients with scores ≥ 8 or < 8 (Pearson’s χ^2^ test). (C) HADS-D scores (Mann-Whitney U test) and percentage of patients with scores ≥ 8 or < 8 (Pearson’s χ^2^ test). (D) Factors associated with HADS total scores ≥16. HADS, Hospital Anxiety and Depression; OR, odds ratio. The severe group was defined as those with fever or suspected respiratory infection, plus one of the following conditions: respiratory rate more than 30 breaths per minute; severe respiratory distress; or oxygen saturation as measured by pulse oximetry less than or equal to 93% on room air.

### HADS scores at the two-year follow-up

Two years after hospital discharge, the median (IQR) HADS score was 5(0-9), and patients with severe disease had a prominently higher median (IQR) HADS score (6 [2-10]) than the nonsevere group (4 [0-8]; *P* < 0.001). A total of 59 patients (5.2%) had HADS scores of at least 16, and the severe group showed a markedly higher proportion of patients with a HADS score of at least 16 (severe vs. nonsevere: 8.1% [32 patients] vs. 3.7% [27 patients]; *P* = 0.001) (Fig. 4A). The median (IQR) HADS-A score was 1 (0-3), and a total of 43 (3.8%) patients had HADS-A scores of at least 8. For the HADS-D, the median (IQR) score was 3 (0-6), and 130 (11.5%) patients had scores of 8 or higher. Similar to the results of HADS total scores, conspicuously higher HADS-A and HADS-D scores were observed in patients with severe disease, along with a higher proportion of patients with scores of at least 8 (Fig. 4B and 4C). Multivariate analysis revealed that older age (OR: 1.04, 95% CI: 1.00-1.08, *P* = 0.03), severe disease during hospitalization (OR: 2.20, 95% CI: 1.28-3.79, *P* = 0.004), and coexisting cerebrovascular diseases (OR: 4.05, 95% CI: 1.84-8.93, *P* = 0.001) were risk factors for HADS total scores of 16 or higher (Fig. 4D and Supplementary Table 4). Furthermore, a positive linear correlation between HADS total scores and CIS-fatigue scores (r=0.43, *P* < 0.001) was seen in a regression analysis (Supplementary Fig. 1), which indicated a potential link between the two symptoms.

## DISCUSSION

The present study reported the long-term symptom burden of older COVID-19 survivors 2 years after hospital discharge and explored the potential associated factors. The research found that 259 patients (21.4%) still reported at least 1 symptom, and common symptoms were fatigue, anxiety, dyspnea, along with a notable proportion of depression measured by the HADS-D subscale. Fatigue, anxiety and chest symptoms often co-occurred at the level of symptom clusters. Altogether, 89 patients (7.7%) had CIS scores of at least 27, for which the relevant risk factors included age and oxygen therapy. In addition, older age, severe disease during hospitalization, and coexisting cerebrovascular diseases were associated with increased risks of HADS total scores of at least 16 (59 patients [5.2%]). Taken together, these findings add to the current knowledge of long-term health outcomes in older COVID-19 survivors.

Growing evidence shows that older adults are at a higher risk for the detrimental health effects of SARS-CoV-2 [17]. Older people face a decline in immunity and the degradation of self-repair function [18, 19]. A reduced abundance of naïve CD8 T cells with decreased expression of antiviral defense genes (i.e., IFITM3 and TRIM22) was identified in aged patients with severe COVID-19 [19]. In the current study, persistent fatigue, anxiety, dyspnea, and depression were found in a proportion of older patients with COVID-19. With the global escalation of the aging process, more special attention and health care are necessary for older people, who seem to be more fragile under the COVID-19 pandemic.

Fatigue was found to be the most common self-reported long COVID symptom in older COVID-19 survivors regardless of disease severity, which was in line with other studies [20, 21]. Previously, Lewis, G. et al. proposed a view that fatigue consisted of both emotional and behavioral components [22]. Morgul E et al. suggested that in the context of the COVID-19 pandemic, the experience of physical and psychological fatigue might be related to preventive restrictions of movement due to isolation and lockdown or have a relationship with depression and anxiety caused by an unprecedented pandemic with unknown duration [23]. Besides, it was suggested that the interaction between SARS-CoV-2 and ACE2 might lead to increased skeletal muscle viral susceptibility and myopathies [24]. These findings may explain the symptom cluster pattern to some extent that fatigue was clustered with myalgia based on their strong correlation and this symptom cluster most frequently co-occurred with anxiety. Additionally, fatigue symptom cluster co-occurred with almost all other disorders including headache, anorexia, and alopecia—similar to another study [25], and old people were previously identified to have an increased risk of longitudinal cognitive decline [26]. These findings may indicate a potential link between long COVID and chronic fatigue syndrome, which usually includes similar symptoms as stated above as well as significant neurocognitive impairment and usually persists for years [26]. Further studies are needed to investigate their relationships and, in the meantime, be alert to the interference of Alzheimer's disease in this particular population.

Anxiety and depression are frequently reported symptoms among individuals as part of post COVID-19 syndromes. In the current study, self-reported anxiety symptoms were identified in 7.4% of survivors, and 3.8% of survivors had HADS-A scores of at least 8. Although no patient reported depression in the self-reported symptom questionnaire, 11.5% had HADS-D scores of 8 or higher. A previous study suggested that the HADS exhibited overall good psychometric properties in evaluating the presence of anxiety and depressive symptoms in previously hospitalized COVID-19 survivors [27], thus the absence of self-reported depression may be due to the lack of self-awareness of depression in older adults with COVID-19. In another study on COVID-19 survivors who had been admitted to the ICU, 14.1% had antidepressant treatments and 18.5% consumed anxiolytics at 6 months after discharge, which also reflected the "real" burden of anxiety and depression symptoms [28]. Therefore, it is important to combine self-evaluation with clinical assessment and the information about antipsychotic consumption in long-term follow-up for the evaluation of psychological burden among older adults with COVID-19.

The fact that 11.5% of patients had HADS-D scores of 8 or higher suggests that depression is another symptom that needs attention, since depressive symptoms may influence neurocognitive functioning. Previously, two separate studies investigated the relationship between depression and neurocognitive functioning in COVID-19 patients and reported that patients with depression performed worse on neurocognitive tests than those without depression [29, 30]. In addition, concerns should be raised regarding whether the depression among COVID-19 survivors is indeed the long-term consequence of the viral infection or just a result of the social and/or economic influence of the pandemic. For example, higher rates of depressive symptoms have been reported in the general population since the onset of the pandemic [31]. In a systematic review containing 11 studies, female, loneliness, poor sleep quality and inferior motor function were confirmed to be risk factors for both depression and anxiety in adults ≥ 60 years old during the COVID-19 pandemic [32]. For external comparison, a previous case-control study in Morocco reported that at 3 months after hospital discharge, COVID-19 survivors aged over 60 years had a higher burden of anxiety and depression (mean value: HADS-A = 16.80; HADS-D = 14.29) than the control group (mean value: HADS-A = 12.58; HADS-D = 10.40) [33]. Another study from France showed that the percentage of anxious and depressive symptoms among COVID-19 survivors discharged from the ICU was 48.2%, which was even slightly lower than that in the control population (50.9%) admitted to the ICU for other reasons at 6 months after discharge [28]. Besides, sensitivity analysis from a systematic review of 33 observational studies on the long-term effects of COVID-19 revealed a decrease in anxiety and depression with longer follow-up time [34]. Therefore, the relatively lower levels of anxious and depressive symptoms in the current study may be due to the impact of cultural influences and longer follow-up time after hospital discharge.

The strengths of this study include the large sample size and a relatively naïve population, who were infected by the original strain of SARS-CoV-2 during the first wave of the COVID-19 outbreak and lacked confounding effects due to vaccination or previous infections with different variants. Besides, we applied the CIS and HADS rating scales to quantify the burden of the most common symptoms among older participants. However, the present study also has some limitations. First, it is noteworthy that patients lost to follow-up were older and presented a higher percentage of coexisting cerebrovascular diseases and chronic kidney diseases compared with enrolled patients, which may introduce some bias. Second, telephone interviews may be informationally biased due to some inaccuracies in understanding and judgment, and the self-evaluation of symptoms can be biased by psychological components and of unclear validity without concomitant physical, biochemical and immunological assessments. Third, the lack of a contemporaneous control group of participants who were never infected with COVID-19 led to the possibility that the observed symptoms stem from or are related to other health problems with aging.

In the future, ongoing follow-up studies are required to better characterize the parallel change in these symptoms and observe the prognosis of long COVID with treatment interventions. Additionally, prioritizing mental health may be suggested for clinicians to promote the long-term resilience of older COVID-19 survivors.

## Supplementary Materials

The Supplementary data can be found online at: www.aginganddisease.org/EN/10.14336/AD.2023.0304.
