# Supplementary material for: Symptom Burden among Older COVID-19 Survivors Two Years after Hospital Discharge
Source: Aging Dis. 2023 Dec 1;14(6):2238–48. doi: 10.14336/AD.2023.0304 (PMC10676794; doi:10.14336/AD.2023.0304)
Supplement: Supplementary file 1 — The Supplementary data can be found online at: www.aginganddisease.org/EN/10.14336/AD.2023.0304. [file AD-14-6-2238-s.pdf]

SUPPLEMENTARY DATA

# Symptom Burden among Older COVID-19 Survivors Two Years after Hospital Discharge

**Ying Zhang[1#], Zhaojie Han[2#], Yang Dai[1#], Yuhui Liu[3, 4#], Qinghua Wang[3, 4#], Lixia Cheng[5, 6], Chuyue Xiong[1], Chao Hou[1], Xinyue Yang[1], Yidan Ye[1], Qian Zhao[1], Naifu Nie[1], Xiangyu Ma[7], Huan Tang[1], Anqiang Zhang[8], Zhenhong Hu[9], Guoqiang Cao[1,4], Paul Jones[10, 11], Jianxin Jiang[8], Ling Zeng[8*], Yong He[1*], Li Li[1, 4*]**

© 2023. Zhang Y et al. Published online at http://www.aginganddisease.org/EN/10.14336/AD.2023.0303

# SUPPLEMENTARY DATA

**Supplementary Table 1.** Symptoms during hospitalization vs. 2-year follow-up according to disease severity.

| Symptoms | Participants (N=1212) | | | Severe (N=422) | | | Nonsevere(N=790) | | | *P* value (2-year Severe vs. Nonsevere) |
|---|---|---|---|---|---|---|---|---|---|---|
| | hospitalization | 2-year | *P* value | hospitalization | 2-year | *P* value | hospitalization | 2-year | *P* value | |
| Any one of the following symptoms | 1151(95.0) | 259(21.4) | <0.001 | 407(96.4) | 107(25.4) | <0.001 | 744(94.2) | 152(19.2) | <0.001 | 0.01 |
| Fatigue | 723(59.7) | 113(9.3) | <0.001 | 255(60.4) | 42(10.0) | <0.001 | 468(59.2) | 71(9.0) | <0.001 | 0.58 |
| Sweating | - | 11(0.9) | - | - | 3(0.7) | - | - | 8(1.0) | - | 0.76[a] |
| Chest tightness | 394(32.5) | 27(2.2) | <0.001 | 144(34.1) | 14(3.3) | <0.001 | 250(31.6) | 13(1.6) | <0.001 | 0.06 |
| Anxiety | - | 90(7.4) | - | - | 43(10.2) | - | - | 47(5.9) | - | 0.01 |
| Myalgia | 337(27.8) | 30(2.5) | <0.001 | 109(25.8) | 14(3.3) | <0.001 | 228(28.9) | 16(2.0) | <0.001 | 0.17 |
| Palpitation | - | 2(0.2) | - | - | 2(0.5) | - | - | 0 | - | 0.12[a] |
| Cough | 834(68.8) | 26(2.1) | <0.001 | 304(72.0) | 9(2.1) | <0.001 | 530(67.1) | 17(2.2) | <0.001 | 0.98 |
| Short of breath | 559(46.1) | 7(0.6) | <0.001 | 220(52.1) | 2(0.5) | <0.001 | 339(42.9) | 5(0.6) | <0.001 | 1.00[a] |
| Dizzness | 35(2.9) | 9(0.7) | <0.001 | 14(3.3) | 4 (0.9) | 0.02 | 21(2.7) | 5(0.6) | 0.002 | 0.73[a] |
| Expectoration | 208(17.2) | 24(2.0) | <0.001 | 91(21.6) | 7(1.7) | <0.001 | 117(14.8) | 17(2.2) | <0.001 | 0.56 |
| Dyspnea | 102(8.4) | 30(2.5) | <0.001 | 68(16.1) | 11(2.6) | <0.001 | 34(4.3) | 19 (2.4) | 0.04 | 0.83 |
| Headache | 28(2.3) | 7(0.6) | <0.001 | 9(2.1) | 1(0.2) | 0.01 | 19(2.4) | 6(0.8) | 0.009 | 0.43[a] |
| Edema of lower limbs | - | 6(0.5) | - | - | 2(0.5) | - | - | 4(0.5) | - | 1.00[a] |
| Taste change | - | 1(0.1) | - | - | 0 | - | - | 1(0.1) | - | 1.00[a] |
| Smell reduction | - | 4(0.3) | - | - | 0 | - | - | 4(0.5) | - | 0.30[a] |
| Sore throat | 63(5.2) | 3(0.2) | <0.001 | 18(4.3) | 0 | - | 45(5.7) | 3(0.4) | 0.06 | 0.56[a] |
| Anorexia | 668(55.1) | 3(0.2) | <0.001 | 234(55.5) | 2(0.5) | <0.001 | 434(54.9) | 1(0.1) | 0.02 | 0.28[a] |
| Diarrhea | 81(6.7) | 7(0.6) | <0.001 | 28(6.6) | 2(0.5) | <0.001 | 53(6.7) | 5(0.6) | 0.62 | 1.00[a] |
| Hemoptysis | 10(0.8) | 0 | 0.002 | 4(0.9) | 0 | 0.12[a] | 6(0.8) | 0 | 0.03[a] | - |
| Nausea | 35(2.9) | 3(0.2) | <0.001 | 12(2.8) | 0 | <0.001 | 23(2.9) | 3(0.4) | <0.001 | 0.56[a] |
| Chill | 23(1.9) | 1(0.1) | <0.001 | 13(3.1) | 0 | <0.001 | 10(1.3) | 1(0.1) | 0.006 | 1.00[a] |
| Vomiting | 30(2.5) | 1(0.1) | <0.001 | 15(3.6) | 0 | <0.001 | 15(1.9) | 1(0.1) | <0.001 | 1.00[a] |
| Alopecia | - | 3(0.2) | - | - | 0 | - | - | 3(0.4) | - | 0.56[a] |

a, Fisher's exact test. The rest of the statistical tests were calculated with the Pearson's $\chi^2$ test.

**Supplementary Table 2.** Symptoms at 1-year follow-up vs. 2-year follow-up according to disease severity

| Symptoms | Participants(N=890) | | | Severe(N=322) | | | Nonsevere(N=568) | | | *P* value (2-year Severe vs. Nonsevere) |
|---|---|---|---|---|---|---|---|---|---|---|
| | 1-year | 2-year | *P* value | 1-year | 2-year | *P* value | 1-year | 2-year | *P* value | |
| Any one of the following symptoms | 437(49.1) | 194(21.8) | <0.001 | 177(55.0) | 81(25.2) | <0.001 | 260(45.8) | 113(19.9) | <0.001 | 0.07 |
| Fatigue | 273(30.7) | 83(9.3) | <0.001 | 115(35.7) | 28(8.7) | <0.001 | 158(27.8) | 55(9.7) | <0.001 | 0.63 |
| Sweating | 174(19.6) | 11(1.2) | <0.001 | 76(23.6) | 3(0.9) | <0.001 | 98(17.3) | 8(1.4) | <0.001 | 0.76[a] |
| Chest tightness | 134(15.1) | 23(2.6) | <0.001 | 64(19.9) | 12(3.7) | <0.001 | 70(12.3) | 11(1.9) | <0.001 | 0.11 |
| Anxiety | 100(11.2) | 51(5.7) | <0.001 | 41(12.7) | 28(8.7) | 0.10 | 59(10.4) | 23(4.0) | <0.001 | 0.004 |
| Myalgia | 74(8.3) | 29(3.3) | <0.001 | 34(10.6) | 13(4.0) | 0.001 | 40(7.0) | 16(2.8) | 0.001 | 0.32 |
| Palpitation | 48(5.4) | 2(0.2) | <0.001 | 19(5.9) | 2(0.6) | <0.001 | 29(5.1) | 0 | <0.001 | 0.13[a] |
| Cough | 48(5.4) | 24(2.7) | 0.004 | 26(8.1) | 9(2.8) | 0.003 | 22(3.9) | 15(2.6) | 0.24 | 0.89 |
| Short of breath | 40(4.5) | 6(0.7) | <0.001 | 22(6.8) | 2(0.6) | <0.001 | 18(3.2) | 4(0.7) | 0.003 | 1.00[a] |
| Dizziness | 38(4.3) | 9(1.0) | <0.001 | 13(4.0) | 4 (1.2) | 0.03 | 25(4.4) | 5(0.9) | <0.001 | 0.73[a] |
| Expectoration | 38(4.3) | 23(2.6) | 0.05 | 21(6.5) | 7(2.2) | 0.007 | 17(3.0) | 16(2.8) | 0.86 | 0.56 |
| Dyspnea | 32(3.6) | 30(3.4) | 0.80 | 17(5.3) | 11(3.4) | 0.25 | 15(2.6) | 19 (3.3) | 0.49 | 0.96 |
| Headache | 21(2.4) | 7(0.8) | 0.008 | 12(3.7) | 1(0.3) | 0.002 | 9(1.6) | 6(1.1) | 0.44 | 0.43[a] |
| Edema of lower limbs | 18(2.0) | 5(0.6) | 0.006 | 10(3.1) | 2(0.6) | 0.02 | 8(1.4) | 3(0.5) | 0.13 | 1.00[a] |
| Taste change | 18(2.0) | 1(0.1) | <0.001 | 8(2.5) | 0 | 0.01 | 10(1.8) | 1(0.2) | 0.006 | 1.00[a] |
| Smell reduction | 16(1.8) | 4(0.4) | 0.007 | 9(2.8) | 0 | 0.006 | 7(1.2) | 4(0.7) | 0.36 | 0.30[a] |
| Sore throat | 8(0.9) | 2(0.2) | 0.06 | 5(1.6) | 0 | 0.12[a] | 3(0.5) | 2(0.4) | 1.00[a] | 0.54[a] |
| Anorexia | 7(0.8) | 2(0.2) | 0.18[a] | 2(0.6) | 1(0.3) | 1.00[a] | 5(0.9) | 1(0.2) | 0.22[a] | 1.00[a] |
| Diarrhea | 5(0.6) | 7(0.8) | 0.56 | 1(0.3) | 2(0.6) | 1.00[a] | 4(0.7) | 5(0.9) | 1.00[a] | 1.00[a] |
| Hemoptysis | 0 | 0 | - | 0 | 0 | - | 0 | 0 | - | - |
| Nausea | 1(0.1) | 3(0.3) | 0.63[a] | 0 | 0 | - | 1(0.2) | 3(0.5) | 0.62[a] | 0.56[a] |
| Chill | 1(0.1) | 1(0.1) | 1.00[a] | 0 | 0 | - | 1(0.2) | 1(0.2) | 1.00[a] | 1.00[a] |
| Vomiting | 1(0.1) | 1(0.1) | 1.00[a] | 0 | 0 | - | 1(0.2) | 1(0.2) | 1.00[a] | 1.00[a] |
| Alopecia | - | 2(0.2) | - | - | 0 | - | - | 2(0.4) | - | 0.54[a] |

a, Fisher's exact test. The rest of the statistical tests were calculated with the Pearson's $\chi^2$ test.

© 2023. Zhang Y et al. Published online at http://www.aginganddisease.org/EN/10.14336/AD.2023.0303

# SUPPLEMENTARY DATA

**Supplementary Table 3.** Logistic regression models to evaluate the risk factors for CIS score ≥27.

| Variables | Univariable ORs(95%CI) | *P* value | Multivariable ORs(95%CI) | *P* value |
|---|---|---|---|---|
| **Age, year** | 1.08(1.05-1.12) | <0.001 | 1.08(1.05-1.11) | <0.001 |
| **Sex, female** | 1.29(0.84-1.99) | 0.25 | 1.20(0.77-1.86) | 0.43 |
| **Severity, vs. no** | 1.52(0.98-2.36) | 0.06 | 1.11(0.70-1.75) | 0.66 |
| **ICU admission, vs. no** | 1.86(0.71-4.89) | 0.21 | | |
| **Oxygen therapy, vs. no** | 2.60(1.29-5.26) | 0.008 | 2.19(1.06-4.50) | 0.03 |
| **Mechanical ventilation, vs. no** | 2.03(0.59-7.03) | 0.26 | | |
| **Cigarette smoking，vs. never** | | | | |
| **Former** | 4.00(1.08-14.79) | 0.04 | | |
| **Active** | 0.86(0.34-2.22) | 0.76 | | |
| **Coexisting disorder, vs. no** | | | | |
| **Hypertension** | 0.71(0.45-1.12) | 0.14 | | |
| **Diabetes** | 1.43(0.86-2.37) | 0.17 | | |
| **Cardiovascular diseases** | 0.57(0.28-1.16) | 0.12 | | |
| **Chronic liver diseases** | 1.35(0.52-3.49) | 0.54 | | |
| **Cerebrovascular diseases** | 2.63(1.28-5.38) | 0.008 | | |
| **Chronic kidney diseases** | 0.92(0.21-3.94) | 0.91 | | |
| **Tumor** | 1.68(0.58-4.89) | 0.34 | | |
| **COPD** | 3.29(1.07-10.15) | 0.04 | | |

Dependent variable: CIS score ≥ 27.
Independent variables: Age, Sex, Severity, Oxygen therapy (excluding mechanical ventilation), Cigarette smoking, Cerebrovascular diseases, COPD.
Abbreviations: ICU, Intensive Care Unit; COPD, Chronic Obstructive Pulmonary Disease.

**Supplementary Table 4.** Logistic regression models to evaluate the risk factors for HADS score ≥16.

| Variables | Univariable ORs(95%CI) | *P* value | Multivariable ORs(95%CI) | *P* value |
|---|---|---|---|---|
| **Age, year** | 1.06(1.02-1.10) | 0.002 | 1.04(1.00-1.08) | 0.03 |
| **Sex, female** | 0.84(0.49-1.42) | 0.52 | 0.64(0.37-1.12) | 0.12 |
| **Severity, vs. no** | 2.32(1.37-3.96) | 0.002 | 2.20(1.28-3.79) | 0.004 |
| **ICU admission, vs. no** | 2.21(0.64-5.80) | 0.15 | | |
| **Oxygen therapy, vs. no** | 2.49(1.14-6.54) | 0.04 | | |
| **Mechanical ventilation, vs. no** | 3.13(0.72-9.59) | 0.07 | | |
| **Cigarette smoking, vs. never** | | | | |
| **Former** | 3.31(0.50-12.69) | 0.13 | | |
| **Active** | 0.45(0.07-1.51) | 0.28 | | |
| **Coexisting disorder, vs. no** | | | | |
| **Hypertension** | 0.88(0.51-1.49) | 0.63 | | |
| **Diabetes** | 1.08(0.54-2.01) | 0.81 | | |
| **Cardiovascular diseases** | 1.38(0.69-2.57) | 0.34 | | |
| **Chronic liver diseases** | 0.38(0.02-1.76) | 0.34 | | |
| **Cerebrovascular diseases** | 4.44(2.02-8.99) | <0.001 | 4.05(1.84-8.93) | 0.001 |
| **Chronic kidney diseases** | 0.67(0.09-4.98) | 0.69 | | |
| **Tumor** | 1.18(0.19-4.02) | 0.83 | | |
| **COPD** | 1.01(0.06-5.01) | 1.00 | | |

Dependent variable: HADS score ≥16.
Independent variables: Age, Sex, Severity, Oxygen therapy (excluding mechanical ventilation), Mechanical ventilation, Cerebrovascular diseases.
Abbreviations: ICU, Intensive Care Unit; COPD, Chronic Obstructive Pulmonary Disease

© 2023. Zhang Y et al. Published online at http://www.aginganddisease.org/EN/10.14336/AD.2023.0303

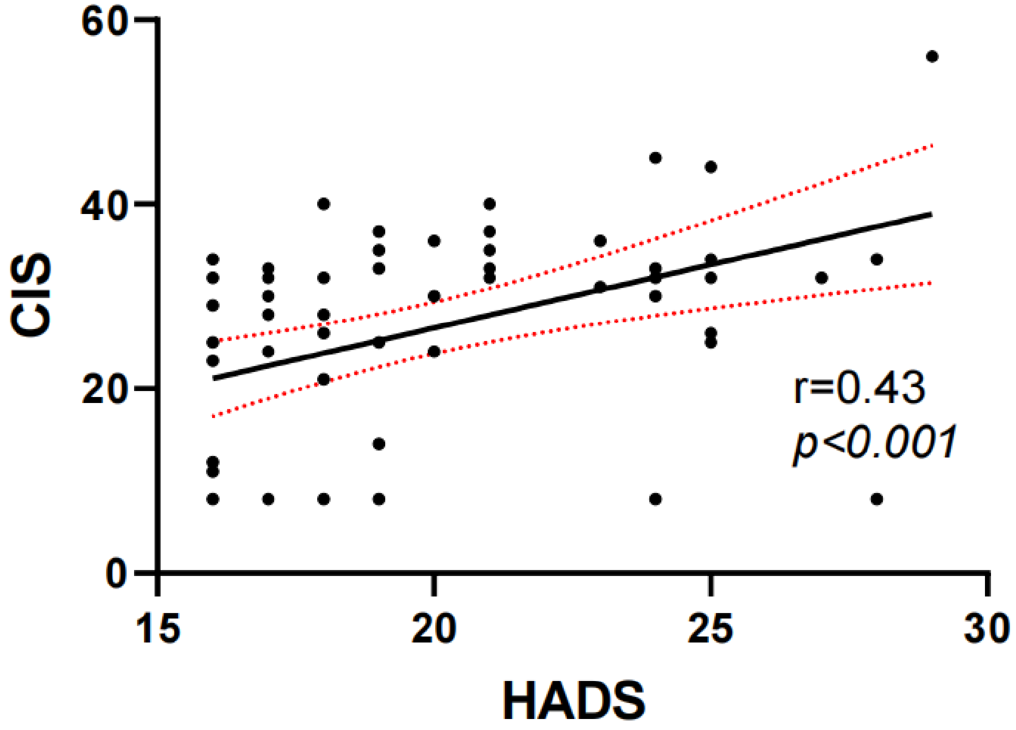


**Supplementary Figure 1. Linear regression of HADS ≥16. Dashed lines represent 95% confidence interval.**

© 2023. Zhang Y et al. Published online at http://www.aginganddisease.org/EN/10.14336/AD.2023.0303
